# Sequence Identification, Recombinant Production, and Analysis of the Self-Assembly of Egg Stalk Silk Proteins from Lacewing *Chrysoperla carnea*

**DOI:** 10.3390/biom7020043

**Published:** 2017-06-13

**Authors:** Martin Neuenfeldt, Thomas Scheibel

**Affiliations:** 1 Lehrstuhl Biomaterialien, Fakultät für Ingenieurwissenschaften, Universität Bayreuth, Universitätsstraße 30, 95440 Bayreuth, Germany; martin.neuenfeldt@bm.uni-bayreuth.de; 2 Forschungszentrum für Bio-Makromoleküle (BIOmac), Bayrisches Geoinstitut, Universität Bayreuth, Universitätsstraße 30, 95440 Bayreuth, Germany; 3 Bayreuther Materialzentrum (BayMat), Fakultät für Ingenieurwissenschaften, Universität Bayreuth, Universitätsstraße 30, 95440 Bayreuth, Germany; 4 Bayrisches Polymerinstitut (BPI), Universität Bayreuth, Universitätsstraße 30, 95440 Bayreuth, Germany; 5 Bayreuther Zentrum für Kolloide und Grenzflächen (BZKG), Universität Bayreuth, Naturwissenschaften I, 95440 Bayreuth, Germany; 6 Bayreuther Zentrum für Molekulare Biowissenschaften (BZMB), Universität Bayreuth, Naturwissenschaften I, 95440 Bayreuth, Germany

**Keywords:** insect silk, qPCR, transcript variants, genome analysis, recombinant proteins, circular dichroism, self-assembly

## Abstract

Egg stalk silks of the common green lacewing *Chrysoperla carnea* likely comprise at least three different silk proteins. Based on the natural spinning process, it was hypothesized that these proteins self-assemble without shear stress, as adult lacewings do not use a spinneret. To examine this, the first sequence identification and determination of the gene expression profile of several silk proteins and various transcript variants thereof was conducted, and then the three major proteins were recombinantly produced in *Escherichia coli* encoded by their native complementary DNA (cDNA) sequences. Circular dichroism measurements indicated that the silk proteins in aqueous solutions had a mainly intrinsically disordered structure. The largest silk protein, which we named ChryC1, exhibited a lower critical solution temperature (LCST) behavior and self-assembled into fibers or film morphologies, depending on the conditions used. The second silk protein, ChryC2, self-assembled into nanofibrils and subsequently formed hydrogels. Circular dichroism and Fourier transform infrared spectroscopy confirmed conformational changes of both proteins into beta sheet rich structures upon assembly. ChryC3 did not self-assemble into any morphology under the tested conditions. Thereby, through this work, it could be shown that recombinant lacewing silk proteins can be produced and further used for studying the fiber formation of lacewing egg stalks.

## 1. Introduction

Oviposition, a unique egg laying behavior exhibited by most species of female lacewings, results in the production of an egg attached to an egg stalk comprised primarily of silk proteins [1]. Silk proteins are typically associated with arthropods, like silkworms or spiders; however lacewings have several interesting and unique aspects to their silk proteins and their processing. For example, in the case of most arthropods spinning a proper silk fiber requires the mechanical pulling of the silk out of a spinneret [2,3]. In contrast, a spinneret is absent in adult lacewings, and the spinning process of its silk occurs completely outside of the animal’s body. First, a droplet of silk dope is secreted, and then a fiber is drawn by lifting their abdomen out of the secreted droplet of silk dope. After subsequent deposition of an egg at the tip of the silk thread, they hold their lifted position for several seconds until the fiber has dried. The resulting egg stalk protects the egg from predators and cannibalism by lifting it from the ground until the larva hatches [1]. This key difference in silk processing, when comparing most arthropods to lacewing silk, indicates that some form of pre-assembly of the proteins in solution must occur. This makes lacewing silk a particularly interesting tool to study the self-assembly behavior of silk proteins.

Structural studies of lacewing egg stalk silk revealed a native cross-beta conformation [4]. The densely-stacked beta sheets are believed to contribute to the bending stiffness of the stalks. Further stretching of egg stalk fibers results in a structural conversion towards parallel beta sheets, which underlies the exceptional extensibility of this protein compared to other fibrous proteins [4,5]. The extent of this permanent non-elastic deformation depends significantly on the relative humidity, and has a maximum value of 380% [6]. There are few other examples of functional cross-beta structures, and this is likely due to the fact that these structures significantly increase the probability of non-triggered protein aggregation [6].

In previous studies on egg stalks from the endemic Australian species *Mallada signata* two major silk proteins, MalXB1 and MalXB2, were identified [6]. These proteins are rich in serine, glycine, and alanine residues, and both comprise repetitive core domains with a 16-residue periodicity, this periodicity giving rise to the cross-beta conformation [6].

Here we identified for the first time sequences of three egg stalk silk proteins and four transcript variants of the common European green lacewing *Chrysoperla carnea* and their natural gene expression pattern. Collecting natural silk in the amounts required to analyze individual silk proteins in more detail is a daunting task, and recombinant production of silk proteins is a popular, well-established and accepted alternative [7,8,9]. Therefore, all three identified proteins were recombinantly produced based on their natural sequence (complementary DNA) and characterized in solution. Two out of three proteins showed self-assembly properties into two- and three-dimensional morphologies which were structurally characterized in more detail.

## 2. Results

Although egg stalk silks from several lacewing species have been investigated in terms of amino acid composition and concerning structural properties of the fibers [4,10], protein sequence information is only available for two egg stalk proteins of the Australian species *M. signata* [6]. In order to evaluate sequence homologies of egg stalks to a related European member of the family Chrysopidae (Figure S7), silk-encoding complementary DNA (cDNA) sequences of *Chrysoperla carnea* were identified using primers based on nucleotide sequences of MalXB1 and MalXB2, respectively. We identified two sequences sharing high identity with MalXB1 and MalXB2, which we named ChryC1 (78.2 kDa; 78% sequence identity with MalXB1; Genbank accession no.: KY906176) and ChryC2 (48.7 kDa; 76% sequence identity with MalXB2; Genbank accession no.: KY906177) (Figures S1 and S2). The amino acid sequences of both proteins comprise repetitive core domains with a 16-residue periodicity, and, like MalXB1, ChryC1 shows two domains interrupted by a short, non-repetitive region. Additionally, four transcript variants of ChryC1 were identified by using the same primers as for the full-length construct (Figure S3).

**Figure 1:**
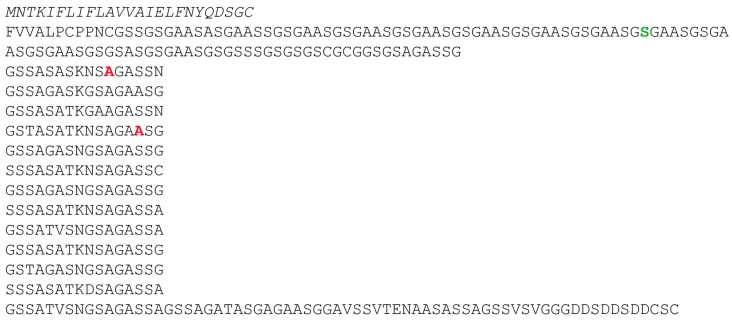
Amino acid sequence of ChryC3 (Genbank accession no.: KY906178). Italics: signal sequence, predicted by SignalP 4.1. Red marked amino acid residues: positions with detected single nucleotide polymorphisms (SNPs) in the cDNA library: gct (Ala) → act (Thr). Green marked amino acid residue: silent SNP (tca → tcg).

The amino acid residues glycine, alanine, and serine are the most abundant residues in silk proteins in general. It is believed that the reason for this conservation is that the residues provide an intermediate hydrophobicity which facilitates the processing of the silk dope towards a fiber [11]. Therefore, we screened for additional putative silk-encoding cDNA sequences using short primers encoding for stretches of glycine, alanine or serine, respectively (Table S1). After identifying a novel sequence fragment which could not be assigned to ChryC1 or ChryC2, we sequenced the full-length cDNA encoding this putative silk protein using the template-switch method by which cDNA molecules of interest are being elongated with a predefined primer sequence at their 3′ end in order to be amplified in a subsequent PCR [12]. The resulting protein, which we named ChryC3 (30.3 kDa; Genbank accession no.: KY906178), shows a similar amino acid composition and the same 16-residue periodicity as the other known egg stalk proteins (Figure 1).

**Figure 2:**
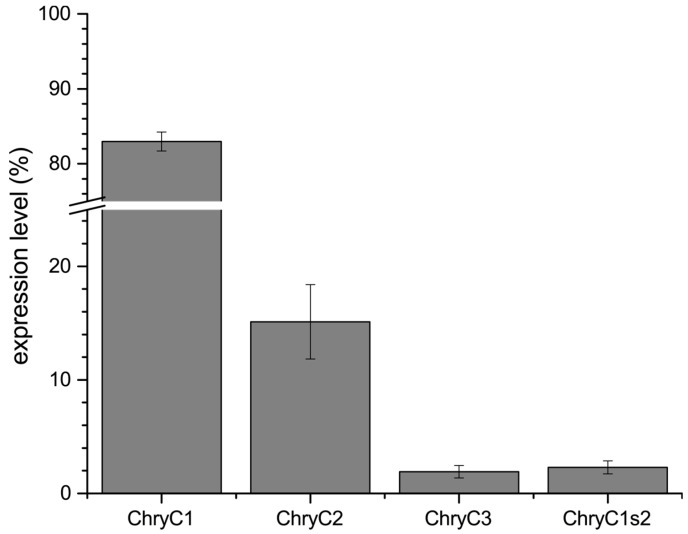
Normalized expression levels of egg stalk genes including the transcript variant ChryC1s2. Error bars indicate standard deviation. The mean expression ratio of ChryC1:ChryC2:ChryC3 is 43:8:1.

Further, we identified the expression ratio between the individual sequences. In the last two decades, pivotal advances regarding sample preparation and analysis of quantitative PCR (qPCR) data have been established, rendering qPCR a highly sensitive method for the determination of gene expression levels [13,14]. For egg stalks of *M. signata*, a significantly higher expression of MalXB1 compared to MalXB2 was observed [6]. In order to determine the expression levels of egg stalk genes of *C. carnea*, qPCR primers were designed to bind to sequences encoding the N-terminal domain of the proteins. For ChryC1, this domain was unaltered in both full-length ChryC1 and all of its transcript variants. Thus, the respective qPCR product represented native ChryC1 and all known transcript variants (Figure S3). In one transcript variant of ChryC1 (namely ChryC1s2; Genbank accession no.: KY906180), the second repetitive domain is deleted. In order to analyze the relative expression level of ChryC1s2, the feature of the deleted domain was exploited on a nucleotide level to design primers which specifically amplify this shortened variant. Analysis of gene expression in colleterial glands showed that ChryC1 exhibited the highest gene expression level, followed by ChryC2 and ChryC3 (Figure 2). The expression level of the transcript variant ChryC1s2 was comparable to that of ChryC3. There was no detected expression of any egg stalk genes in male lacewing and, therefore, they acted as a negative control group in this experiment (Figure S4). To our best knowledge, this is the first quantification of transcript variants of lacewing silk by qPCR.

**Figure 3:**
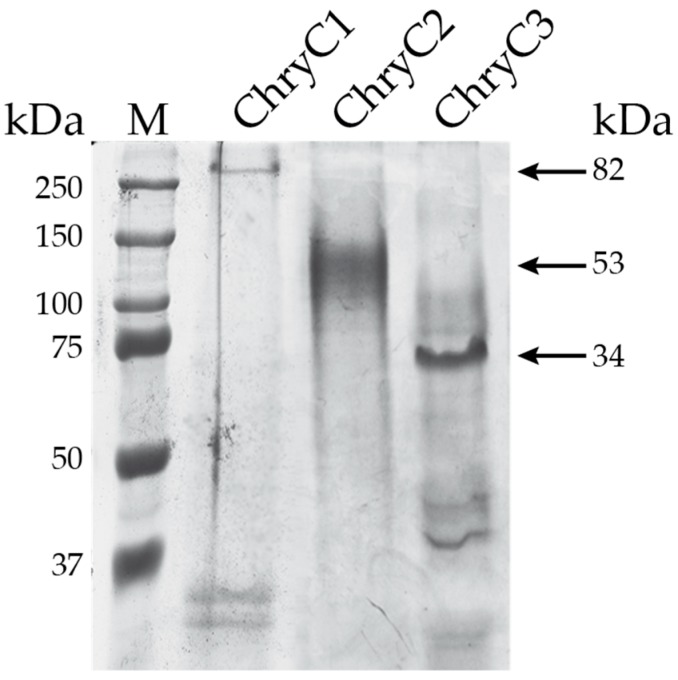
SDS-PAGE of purified egg stalk proteins. Due to their amino acid composition, all proteins run slower than expected for their theoretical molecular weight given next to the arrows. However, such a feature is often seen for the assay for silk proteins due to an insufficient interaction with SDS [16]. First lane shows the marker (M).

For recombinant protein production, the native nucleotide sequences encoding the three full-length egg stalk proteins were cloned into an *Escherichia coli* expression vector using a PCR cloning strategy. The advantage of this technique was there was no need for restriction enzymes to prepare the constructs [15]. Initially for ChryC1 a major drop in protein yield was observed at high cell density fermentation and, therefore, the fermentation conditions had to be adjusted to obtain the highest possible protein yield. Induction of gene expression at cell densities between OD_600 nm_ of 3.0 and 3.5 (for 4 h at 30 °C) led to the most effective production of protein with yields of up to 109 mg of protein per 100 g of wet cell mass. All produced proteins were soluble and purified by a strategy of step-wise protein precipitation (Figure 3).

**Figure 4:**
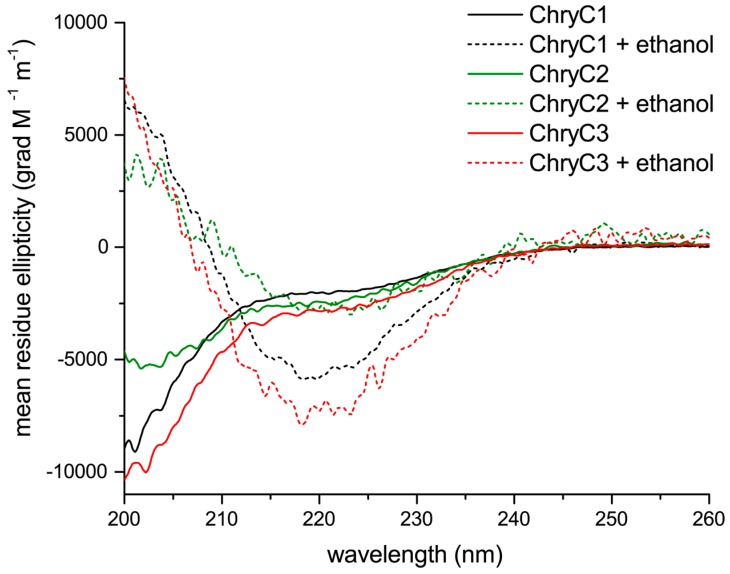
Circular dichroism spectra of ChryC1, ChryC2, and ChryC3 in 10 mM NH_4_HCO_3_, in the absence or presence of four volumes of 100% ethanol (pro analysis; p.a.), respectively.

Circular dichroism (CD) analysis revealed a mainly random coil structure indicated by local minima below 200 nm, with some alpha helical content in aqueous solution (Figure 4) [17]. Interestingly, beta-sheet-rich structures could be induced in the presence of ethanol, indicated by local minima at 218 nm and local maxima below 200 nm (Figure 4).

**Figure 5:**
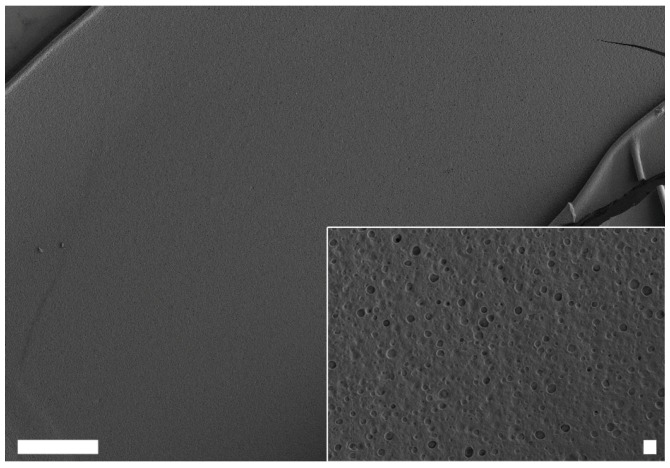
Scanning electron microscopy (SEM) images of self-assembled ChryC1 films. Scale bars: 100 µm and 2 µm (inset), respectively.

With the protein produced and the ability to assemble confirmed, it had to be determined if any of the three identified silk proteins showed self-assembly properties, as it was hypothesized this would be necessary for egg stalk fiber formation. At concentrations above 6 mg·mL^−1^ ChryC1 self-assembled into sheet-like film structures (Figure 5). Measurements by Fourier transform infrared spectroscopy (FTIR) and subsequent Fourier self-deconvolution (FSD) of the dried ChryC1 films revealed that these films comprised a high content of beta-sheets, nearly 50% (Table 1, Figure S6). In order to exclude this structural composition from being induced by the drying process, ChryC1 films were incubated in D_2_O and measured in the wet state as a control and the distribution of secondary structure elements were shown to be unchanged (Table 1).

**Figure 6:**
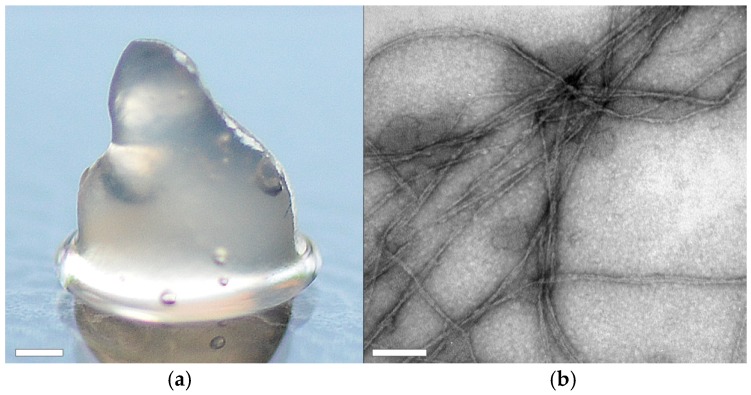
Morphology of a self-assembled ChryC2 hydrogel. (**a**) Photograph of aChryC2 hydrogel. Scale bar: 1 mm; (**b**) Transmission electron microscopy imaging revealed the morphology of the underlying fibrils was 5 nm in diameter. Scale bar: 100 nm.

ChryC2, in contrast, self-assembled into hydrogels above concentrations of 1 mg·mL^−1^. Analysis using transmission electron microscopy (TEM) revealed the presence of nanofibrils in this hydrogel (Figure 6b) which comprise significant beta sheet structure content, as confirmed by CD analysis (Figure S5).

ChryC3 did not show any self-assembly properties under the tested conditions.

**Figure 7:**
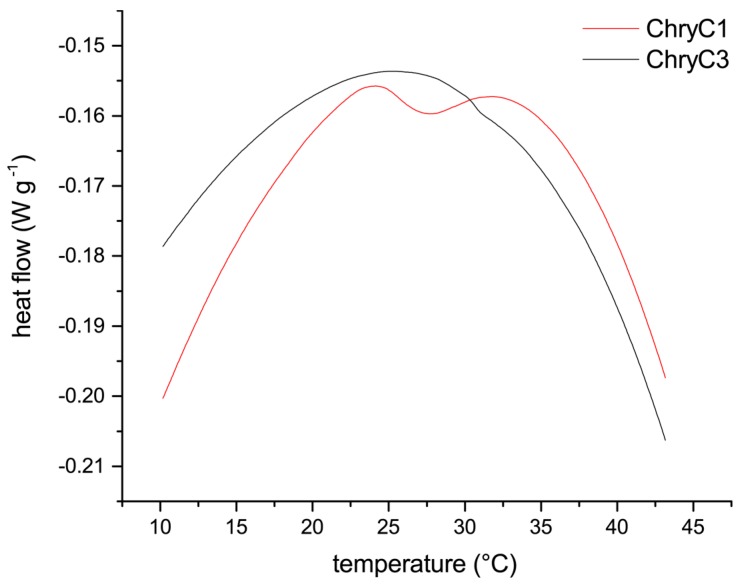
Differential scanning calorimetry measurements of ChryC1 and ChryC3 solutions. For ChryC1, the local minimum of the heat flow at 27.3 °C indicates the transition point for the LCST behavior.

Since self-assembly of proteins into films in fully-aqueous solution is non-typical for proteins, soluble ChryC1 was analyzed in more detail to gain insights into the mechanism of its self-assembly, which revealed a lower critical solution temperature (LCST) behavior resulting in turbid ChryC1 solutions above a distinct temperature. Differential scanning calorimetry (DSC) measurements determined a transition point at 27.3 °C (Figure 7). The LCST behavior could be confirmed upon cooling down the sample, since the agglomeration was completely reversible, as confirmed by ultracentrifugation and subsequent protein concentration determination (data not shown). Since ChryC2 was prone to gelation, this protein was not suitable for DSC measurements. However, heating of low-concentration ChryC2 solutions (<1 mg·mL^−1^) up to 50 °C did not result in any visible turbidity as observed for ChryC1 solutions in the same concentration regime (data not shown). ChryC3 did not exhibit any LCST behavior (Figure 7), which independently confirmed that this protein variant is not able to self-assemble.

**Figure 8:**
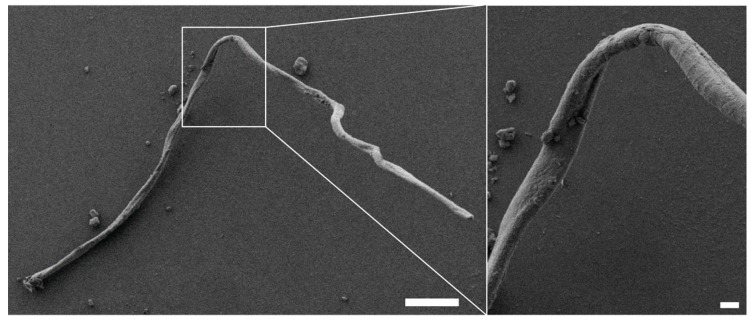
SEM image of a ChryC1 fiber, assembled after addition of four volumes of 100% ethanol (p.a.) to an aqueous solution of ChryC1. The image on the right depicts a magnification of the white frame on the left; scale bars: 100 µm (left) and 10 µm (right), respectively.

Further analysis of the changes in protein structure upon the addition of ethanol revealed that assembly of CryC1 into microscopic fibers could then occur below the LCST. Subsequent scanning electron microscopy (SEM) analysis revealed fiber diameters of 13 µm and smooth surfaces (Figure 8).

## 3. Discussion

In 2009, 50 years after X-ray diffraction studies were first used to identify cross-beta structures in egg stalk fibers from lacewings, the first protein sequence of lacewing silk was identified [6]. In this study, further sequences were identified from the European *C. carnea* and named ChryC1 and ChryC2. These proteins were shown to exhibit a high sequence identity, comparable to the published MalXB1 and MalXB2 of the Australian *M. signata*. The homologous sequences of the two Australian versus European lacewing silks mainly differed regarding the length of individual domains rather than the overall structure of the consecutive domains. For instance, ChryC2 comprises a much shorter N-terminal domain than MalXB2 (63 vs. 152 amino acid residues), yet the repetitive core domains of both proteins exhibit a 16-residue periodicity with a pronounced positive net charge. Interestingly, a further egg stalk silk protein (ChryC3) could be identified, and was shown to be unique compared to the other silk protein variants. Remarkably, all identified sequences, including transcript variants, further shared a motif consisting of cysteine and aspartate residues (in certain variations) at the carboxyl-terminus of their C-terminal domain. The functional role of this motif remains unclear, however, it has already been suggested that cystine cross-links might contribute to the rigidity in egg stalk fibers [6].

Recombinant expression of non-codon-optimized genes (i.e., cDNA comprising the natural sequence) was possible in *E. coli*, which is generally considered to be challenging especially for large and/or highly repetitive sequences [18]. The individual silk proteins considerably differed in their properties, especially in terms of self-assembly. In a concentration-dependent manner, ChryC1 self-assembled into water-insoluble film structures with high beta sheet content and exhibited LCST behavior, whereas ChryC2 formed hydrogels. In contrast, ChryC3 did not show any self-assembly properties, and it is likely that this is a subordinate silk protein; however the exact function cannot be speculated from these results.

All protein variants in aqueous solution could be converted from random coil to beta sheet conformation by adding ethanol to the solvent, which is in good agreement with observations from other silk systems [19]. However, the ethanol-induced self-assembly of ChryC1 fibers in the mm-length regime was unexpected and seems to be unique amongst silk proteins. This supports the hypothesis that the silk fiber processing of lacewing silks most likely depends on triggered self-assembly, and not on shear stress, as in organisms which use a spinning apparatus (spiders, silk worms).

In general, it is assumed that a pre-ordered state of proteins in solution is a necessary condition for natural fiber processing [2]. Interestingly, all three identified proteins of *C. carnea* egg stalks showed an intrinsically disordered structure in solution, but a completely different assembly behavior. The study of recombinant silk proteins is the basis for the determination of the mechanism behind controlled formation of natural egg stalks.

## 4. Materials and Methods

### 4.1. Materials

Chemicals were obtained from Carl Roth (Karlsruhe, Germany) if not stated otherwise. Double-distilled water was prepared using a Millipore system from Merck (Darmstadt, Germany). For dialyses, membranes were used with a molecular weight cut-off of 6–8 kDa (Spectrum Laboratories, Rancho Dominguez, CA, USA). DNA purification and ligation with the pGEM-T vector system was performed according to the manufacturer’s protocol (Promega, Madison, WI, USA). For PCR, Taq polymerase (New England Biolabs, Ipswich, MA, USA) was used if not stated otherwise. Synthetic oligonucleotides for PCR and qPCR were obtained from Microsynth (Balgach, Switzerland) and Eurofins (Ebersberg, Germany), respectively (Table S1). The used cloning strain was *Escherichia coli* DH10B (Novagen, Madison, WI, USA). Green lacewings of species *Chrysoperla carnea* were obtained from Sautter and Stepper (Ammerbuch, Germany).

### 4.2. *Chrysoperla carnea* Silk Gland Partial Transcriptome Sequencing

Colleterial glands of six female lacewings were dissected, immediately transferred into liquid nitrogen and disrupted with mortar and pestle. ChryC1 and ChryC2, RNA isolation and cDNA synthesis was performed using the SV Total RNA Isolation System (Promega, Madison, WI, USA) and the RevertAid First Strand cDNA Synthesis Kit (Thermo Scientific, Waltham, MA, USA), respectively, according to the manufacturer’s protocol. For cDNA synthesis, 10 pmol of a modified oligo-d(T)_18_ primer were used. After the reaction was terminated, RNA was hydrolyzed by adding 3 µmol NaOH and incubation at 95 °C for 5 min, followed by neutralization with HCl. Subsequent amplification was conducted by PCR under the following conditions (used primers: see Table S1: PCR for TA-cloning): 95 °C for 2 min, 30 cycles at 95 °C for 30 s, 36.3 °C for 30 s, 68 °C for 3 min, and final extension at 68 °C for 5 min using a MyCycler from BioRad (Hercules, CA, USA). Additionally, formamide was added after 17 cycles at 0.5% (*v*/*v*) final concentration. After DNA purification, PCR products were ligated with pGEM-T vector and transformed into *E. coli* according to the manufacturer’s protocol. After colony PCR screening, plasmids with inserts were sequenced.

RNA of ChryC3 was obtained by dissecting colleterial glands of 90 female lacewings, as described above. RNA isolation was performed using the Oligotex system (Qiagen, Hilden, Germany) according to the manufacturer’s protocol. cDNA was synthesized by the template switch method, as described previously [12]. Briefly, 290 ng of poly-A+ mRNA were mixed with 20 pmol of sequence-specific primer ChryC3_cDNA, 20 pmol of template-switch primer and 10 nmol of deoxynucleotides (dNTPs) (Table S1). After heating at 65 °C for 5 min, all components of the Maxima H Minus Reverse Transcription system (Thermo Scientific) were added according to the manufacturer’s protocol, and the reaction was performed at 50 °C for 30 min and then terminated at 85 °C for 5 min. RNA was then hydrolyzed by adding 50 nmol NaOH and incubation at 68 °C for 30 min, followed by neutralization with HCl. Subsequently, 15 µL of cDNA were used for amplification by DreamTaq (Thermo Scientific) at Touchdown PCR conditions (used primers: “Anchor forw” and “ChryC3 rev”, see Table S1): 95 °C for 1 min, 5 cycles of 95 °C for 30 s, 56 °C (1 °C decreased at each cycle) for 30 s, 72 °C for 3 min, 30 cycles at 95 °C for 30 s, 50 °C for 30 s, 72 °C for 3 min. After preparative agarose gel electrophoresis, the 1.3 kb DNA band was excised from the gel, purified, ligated with pGEM-T vector, and transformed into *E. coli* as described above. After colony PCR screening, plasmids with inserts were sequenced. The signal peptide was predicted using SignalP 4.1 [20].

The elongation factor 1α (EF-1α) represents a well-established reference gene for qPCR [21]. Initially, in order to be able to design optimal qPCR primers for EF-1 α, this gene of *C. carnea* was partially sequenced. Therefore, genomic DNA was isolated during RNA preparation for qPCR: after the lysate of colleterial glands passed the DNA column of the innuPREP RNA Mini Kit (see 4.3), the column was treated with the DNA purification system starting with a washing step. A 1.9 kb fragment of EF-1α was amplified by PCR with primers designed based on the respective sequence of the related species *Chrysopa perla* (Genbank accession no.: JQ519512.1) [22]. In order to determine the position of the intron segment, PCR was further conducted with cDNA as template. After preparative agarose gel electrophoresis, both products were treated as described above (Genbank accession no. of mRNA fragment: KY906181; Genbank accession no. of genomic DNA fragment: KY906182).

### 4.3. Quantitative PCR

Colleterial glands of six female lacewings and abdomen of five male lacewings were dissected and immediately transferred into liquid nitrogen. The frozen tissue was disrupted in Eppendorf tubes filled with liquid nitrogen using polypropylene pellet pestles from Sigma-Aldrich (St. Louis, MO, USA). RNA isolation was performed using the innuPREP RNA Mini Kit from Analytik Jena (Jena, Germany) according to the manufacturer’s protocol. cDNA synthesis was performed as described above for ChryC1 and ChryC2, yet 100 pmol of random hexamers were used for each sample instead of the modified oligo-d(T)_18_ primer, and subsequent RNA hydrolysis was omitted. Aliquots of all cDNA samples were stored at −80 °C until being used for qPCR.

qPCR was conducted using the innuMIX qPCR MasterMix and the qTOWER 2.0 (Analytik Jena,) according to the manufacturer’s protocol. Every cDNA sample was tenfold diluted prior to analysis, and 4 pmol of each primer were used at a reaction volume of 20 µL. The qPCR program comprised a melting step at 95 °C for 2 min, 30 cycles at 95 °C for 5 s, 65 °C for 20 s, and a melting ramp from 60 to 95 °C. The heating rate was 5 °C·s^−1^. Every sample was analyzed as triplicate; the no-template controls (NTC) were analyzed as duplicate. Additionally to the determination of the melting temperatures of the resulting qPCR products (Table S2), the specificity of amplification was confirmed by 1.5% (*w*/*v*) agarose gel electrophoresis (Figure S4). Based on the raw data, the actual PCR efficiencies and C_q_-values were calculated using the software LinRegPCR [13].

### 4.4. Cloning and Expression of Silk Sequences

All signal-peptide-free (but otherwise full-length native) encoding sequences of ChryC1–3 were cloned into pET28a(+) from Novagen (Madison, WI, USA) by restriction-free cloning [15]. The chosen vector provided an N-terminal His6-tag to each encoding sequence. All proteins were recombinantly produced in *Escherichia coli* BL21-Gold (DE3) pLysS (Novagen) cultured at 30 °C in a 5 L BIOSTAT B plus fermenter (Sartorius, Goettingen, Germany) using lysogeny broth (LB) Lennox medium additionally containing 24 mM (NH_4_)_2_HPO_4_, 35 mg·L^−1^ kanamycin and 0.001% (*v*/*v*) Breox FMT 30 antifoam (Cognis, Monheim, Germany). Expression was induced by adding 0.5 mM isopropyl-β-d-thiogalactopyranoside (IPTG) at an optical density (600 nm) between 3.0 and 3.5. Cells were harvested after expression for 4 h at 30 °C and suspended in buffer (50 mM TRIS/HCl, 100 mM NaCl, pH 8). Recombinant protein production was confirmed by comparing protein composition in *E. coli* cells before and after gene expression induction by SDS-PAGE (data not shown).

### 4.5. Protein Purification

ChryC1 and ChryC3 were purified as previously described for the recombinant protein N[AS]_8_C with slight modifications [23]. Briefly, harvested cells were resuspended in 50 mM TRIS/HCl, 100 mM NaCl, pH 8.0 and after cell disruption, the lysate was treated at 80 °C for 20 min. After centrifugation (20,000× *g* for 20 min), the supernatant was acidified with acetic acid to pH 4.2 and incubated for 2 h. After centrifugation, the supernatant was neutralized with NaOH to pH 8.0 and fractionally precipitated using (NH_4_)_2_SO_4_ (0.8 M and 1.5 M). The pellet was washed with 1.6 M (NH_4_)_2_SO_4_, 25 mM sodium phosphate, pH 8.0 including sonication using a Sonopuls HD3200/KE76 ultrasonicator (Bandelin, Berlin, Germany) until no more DNA was detectable in the washing buffer (monitored using a NanoDrop 1000 (Thermo Scientific)). The final pellet was solubilized in 6 M guanidinium thiocyanate (GdmSCN) and dialyzed against 10 mM NH_4_HCO_3_. Following centrifugation, the supernatant was lyophilized.

Since ChryC2 did not sufficiently precipitate in the presence of (NH_4_)_2_SO_4_, the purification protocol was slightly modified: after heat treatment and acidification, the neutralized supernatant was precipitated in the presence of 0.3% (*w*/*v*) polyethylenimine (PEI; 25,000 g∙mol^−1^) (Sigma-Aldrich,) and centrifuged. After addition of 0.5% (*w*/*v*) sodium dodecylsulfate (SDS) and centrifugation, ChryC2 was precipitated by adding 0.8 volumes of acetone (p.a.) to the supernatant and incubating for 1 h at −20 °C. The resulting pellet was washed with ice-cold DNA washing buffer as described above. ChryC2 was selectively solubilized by adding 4 M urea, 20 mM TRIS/HCl, pH 8.0, and incubated for 16 h at room temperature under constant shaking. After centrifugation and subsequent dialysis of the supernatant against 10 mM NH_4_HCO_3_ the resulting solution was lyophilized.

The extinction coefficient of ChryC2 (18,450 M^−1^·cm^−1^) was calculated using ProtParam [24]; the extinction coefficients of ChryC1 (18,460 M^−1^·cm^−1^) and ChryC3 (11,390 M^−1^ cm^−1^) were determined by gravimetric analysis, since their predicted values were << 10,000 M^−1^ cm^−1^ and not reliable. Since the proteins were not accessible to matrix-assisted laser desorption/ionization (MALDI-TOF), their purity was confirmed by SDS-PAGE as previously described [25]. Additionally, dot blot analysis of the purified proteins confirmed the presence of the His-tag (data not shown).

### 4.6. Secondary Structure Analysis (CD and FTIR)

Far-UV CD spectra were recorded using a J-815 CD spectrometer (Jasco, Gross-Umstadt, Germany) at 20 °C with a scanning interval of 0.1 nm, a digital integration time of 1 s, a bandwidth of 1 nm, a scanning speed of 50 nm·min^−1^, and three accumulations. All measurements were conducted using 4.1–7 µM protein solutions in 10 mM sodium phosphate, pH 7.5 in quartz cuvettes with 1 mm path length; four volumes of 100% ethanol (p.a.) were added to each protein solution to test its impact, and spectra were recorded after incubation for 16 h at room temperature.

FTIR spectra of ChryC1 films were taken using a Tensor 27 spectrometer (Bruker, Billerica, MA, USA). Spectra were measured by attenuated total reflection (ATR) with a resolution of 4 cm^−1^ and averaging of 120 scans. Individual secondary structure elements were determined by FSD of the amide I band (1590–1720 cm^−1^). Band assignments were made according to Hu et al.[26]. The presented data reflect mean values ± standard deviation derived from two films each; each sample was tested at three spots.

### 4.7. Differential Scanning Calorimetry

Differential scanning calorimetry experiments were performed using the DSC 1 STARe System (Mettler-Toledo, Columbus, OH, USA). Measurements were conducted using protein concentrations of 124 µM (ChryC1) and 490 µM (ChryC3), respectively, at a heating rate of 5 K·min^−1^.

### 4.8. Self-Assembly of ChryC1 and ChryC2

ChryC1 (6 mg·mL^−1^) was dissolved in 6 M GdmSCN and dialyzed against 10 mM NH_4_HCO_3_. Any protein aggregates were removed by ultracentrifugation (186,000× *g* for 45 min), and the resulting supernatant was concentrated by dialysis against 20% (*w*/*v*) poly(ethylene glycol) (20,000 g·mol^−1^), 10 mM NH_4_HCO_3_ at 4 °C. Resulting protein assemblies were washed with water and dried at room temperature. In order to exclude artefacts caused by drying, films were prepared and washed 10 times with D_2_O directly after dialysis and measured in the wet state.

For fiber assembly, a ChryC1 solution (2 mg·mL^−1^) was prepared as described above including ultracentrifugation. The resulting supernatant was mixed with 4 volumes of 100% ethanol (p.a.) by inverting the sample.

For hydrogel formation, ChryC2 (3 mg·mL^−1^) was dissolved in 6 M GdmSCN and dialyzed against 10 mM NH_4_HCO_3_. The resulting hydrogel was used for TEM analysis and, in order to increase stability, concentrated to a tenth part of its volume by dialysis against 20% (*w*/*v*) poly(ethylene glycol) (20,000 g·mol^−1^), 10 mM NH_4_HCO_3_ at 4 °C prior photographic documentation.

### 4.9. Electron Microscopy

Samples for SEM were sputter-coated with 1.3 nm of platinum using a 208HR sputter coater (Cressington, Watford, UK) and subsequently analyzed using a 1540 EsB Crossbeam (Zeiss, Oberkochen, Germany) at 2 kV.

Assembled ChryC2 fibrils were diluted to 0.5 mg·mL^−1^, and 5 µL of the dilution were applied on supports (Pioloform-carbon-coated 100-mesh copper grids (Plano GmBH, Wetzlar, Germany)), incubated for 5 min, washed with 5 µL of double-distilled H_2_O, and fibrils were negatively stained for 2 min using 5 µL of 2% uranyl acetate solution. After washing with 5 µL of water, the sample was allowed to dry for 24 h at room temperature before imaging. TEM imaging was performed using a JEM-2100 (JEOL, Tokyo, Japan) operated at 80 kV. Images were recorded using an UltraScan 4000 camera (Gatan, Pleasanton, CA, USA) and Gatan Digital Micrograph software (version 1.83.842).

## Figures and Tables

**Table 1:** Content of secondary structures in ChryC1 films as evaluated by Fourier self-deconvolution. The data represent mean values obtained from two protein films ± standard deviation. All values are rounded to whole numbers.

	Dry Film	Wet Film
Secondary Structure	Fraction (%)	Fraction (%)
Side chains	5 ± 1	11 ± 1
β-sheets	48 ± 2	47 ± 1
α-helices	7 ± 0	5 ± 0
Random coils	24 ± 1	22 ± 0
Turns	17 ± 1	15 ± 1

## Supplementary Materials

The following items are available online at http://www.mdpi.com/2218-273X/7/2/63/s1, Table S1: Overview of synthetic oligonucleotides used in this study, Table S2: Melting temperatures (T_m_) of qPCR products amplified in this study, including standard deviation (s.d.), Figure S1: Protein sequence of ChryC1, Figure S2: Protein sequence of ChryC2, Figure S3: Protein sequences of all found transcript variants of ChryC1, Figure S4: 1.5% (w/v) agarose gel of qPCR products, Figure S5: CD spectrum of self-assembled ChryC2 fibrils, Figure S6: Fourier self-deconvolution (FSD) of FTIR spectra of self-assembled ChryC1 films, Figure S7: Phylogenetic tree of lacewings according to NCBI taxonomy.
