# Supplementary material for: Sequence Identification, Recombinant Production, and Analysis of the Self-Assembly of Egg Stalk Silk Proteins from Lacewing Chrysoperla carnea
Source: Biomolecules. 2017 Jun 13;7(2):43. doi: 10.3390/biom7020043 (PMC5485732; doi:10.3390/biom7020043)
Supplement: Supplementary file 1 [file biomolecules-07-00043-s001.pdf]

Supplementary Information:

Table S1. Overview of synthetic oligonucleotides used in this study.

**cDNA synthesis**	
Mod. oligo-d(T)18	TGGTTTTTTTTTTTTTTTTTT
ChryC3_cDNA	TTATCAATAGCAGTTTCAGTGAAAAATTTAACAAGAGCAGTCATCAGAATCATCAGAATC
Template-switch	AAGCAGTGGTATCAACGCAGAGTCTCGAG(GGG)
**PCR for TA-cloning into pGEM-T vector**	
ChryC1 forw	GGGTGCAATGTGATC
ChryC2 forw	AATTATAATATTTGGAGTAATG
Poly-Ala-stretch	GCAGCAGCAGCGGC
As reverse-primer, modified oligo-d(T)18 was used for both ChryC1 (including all transcript variants), ChryC2, and the first identified fragment of ChryC3.	
Anchor forw (for ChryC3)	AAGCAGTGGTATCAACGCAGAG
ChryC3 rev	GTACCAGTACTCGAGTGCATTTTCAG
**Restriction-free cloning of ChryC1-3 into expression vector**	
ChryC1_forw	GGACAGCAATGGGGTCGCGGATCCATGGGTGGTTGCAATGTGATCGCCTACCCAAC
ChryC1_rev	GGTGCTCGAGTGCGGCCGCAAGCTTTCATTAACAAGAACAATCATCAGAATCATCAG
ChryC2_forw	GGACAGCAATGGGGTCGCGGATCCATGGGTAATTATAATATTTGGAGTAATGTGAACG
ChryC2_rev	GTGGTGCTCGAGTGCGGCCGCAAGCTTTCATTAACATTCATAATCGTAATAATCGTC
ChryC3_forw	GGACAGCAATGGGGTCGCGGATCCATGGGTTTTGTGGTCGCTTTACCATG
ChryC3_rev	GCTCGAGTGCGGCCGCAAGCTTTCATTAACAAGAGCAGTCATCAGAATCATC
**Sequencing of elongation factor 1 alpha (EF-1α)**	
EF-1a_gen_f	GGGTGTCAAACAATTGATTGTCG
EF-1a_gen_r	ACCAGCTACGTATCCTCTTCG
**Colony PCR**	
universe(pUC)	AGGGTTTTCCCAGTCACGACGTT
reverse(pUC)	GAGCGGATAACAATTTCACACAGG
**qPCR**	
qPCR-EF-f	GTCTTCCTCTCCAGGATGTCTAC
qPCR-EF-r	AGCGAATACTACAACCATACCTGG
qPCR-C1-f	GGATCAGGTTCAGCAAGTTCAGG
qPCR-C1-r	AAGCTGCTCCAGATGCTGCAG
qPCR-C1s2-f	ATCAACTGCGACTGCAAGTAAAGAC
qPCR-C1s2-r	ATGCTCCCGATGCAGCTGAG

qPCR-C2-f	TGGATCATCAGCTTCCGGTTCG
qPCR-C2-r	TCCCGAACCGGATGAGGAGC
qPCR-C3-f	TCCACCTAATTGTGGTTCATCAGG
qPCR-C3-r	ACGCTGCGCCTGAACCAGAG

All sequences are indicated according to 5′-3′ nomenclature. RNA-nucleotides are given in parentheses. "qPCR-C1-f" and "qPCR-C1-r" were designed to bind at a conserved region encoding the N-terminal domain of ChryC1, being able to amplify both the full-length construct of ChryC1 and all known transcript variants of ChryC1. In order to prevent both unspecific binding of qPCR primers to competing silk-encoding proteins and formation of primer-dimers, all qPCR primers were analyzed using bioinformatics tools (NCBI primer-BLAST and OligoAnalyzer 3.1). First partial sequencing of ChryC3 was achieved by PCR using "Poly-Ala-stretch" as forward and "Mod. oligo-d$(T)_{18}$" as reverse primers, respectively.

Table S2. Melting temperatures (T_m) of qPCR products amplified in this study, including standard deviation (s.d.).

sample	average T_m [°C]	s.d.
cDNA female EF-1a	79.0	0.1
cDNA male EF-1a	79.1	0.06
cDNA female ChryC1	85.5	0.17
cDNA female	83.3	0.17
cDNA female ChryC2	85.6	0.06
cDNA female ChryC3	86.8	0.06

GCNVIAYPTASCGDSGSGSGSASSGAASGAASGSGAASGSGAASGSGSAAASGAASGSGSAAGSGAASGSGSAAGSG
AASGSGSAAGSGAASGSGSAAGSGAASGSGSGSSSSGSSSSGSSGGCGGGSGSASSG
GSSASATKNSAGASSN
GSSAGASNGSAGASSG
GSSASATKNSAGASSG
SSSAGASNGSAGASSC
GSSATATKNSAGASSG
GSTAGASNGSAGASSG
GSSSSATKNSAGASSN
GSSAGASNGSAGASSG
GSSSSATQNSAGASSN
GSSAGASNGSAGASSG
GSSSSATQNSAGASSN
GSSAGASNGTAGASSG
GSSSSATKESAGASSN
GSTATASKDSAGASSG
GSSVGATASGAGAASGGSVSSATKNSSAASSQGSSVSISNGVVSAASNGATTSAGAGSASSASGGSSANVGG
        GSASGSSN
GATSSANGSSASGSSG
GSSSSAGAGSASGSSG
NSSSSASGNTASGSSG
DSSSSAGSGTASGSSG
GATSTAGSGSASGSSG
SSSSSAGSGSASGSSG
NSSSSASGGTASGSSN
GATSSAGSGTASGSSG
GSSSSAGSGTASGSSG
DSSSSAGSGSASGSSG
GATSSAGSGSASGSSG
NSSSSAGSGSASGSSG
DSSSSAGSGSASGSSG
GATSTAGSGSASGSSG
NSSSSAGSGSASGSSG
DSSSSAGSGSASGSSG
GASSSAGSGSASGSSC
GSTSGASSGSASGSSG
GSSSSAGSGSASGASG
GSSSAAGSGSASGSSG
GSTSGASCGSASGSSG
DSSSSAGSGSASGSSG
GASSSAGSGSASGSSG
GSTSGASSGSASGSSG
GSSSSAGSGSASGASAGSSSAAGSGSASGSSGGSSSGASSGSSDGCGSGGSSGAASGAASGSGSASGSGAASGSGAA
SGSGAASGSGAASGSGAASGSGAASGSGAASGSGSASGLGSAASSGAASSSGSAAGSGSASGSGSAASSGAASSSGS
AAGSGSASVSGSSDDSDDCSC

Figure S1. Protein sequence of ChryC1. Genbank Accession No.: KY906176.

```
NYNIWSNVNAHPTNCDNSGGSSGSSASGSGAASGSGSASGSGAASGSSSGSGSSSGSGCGSGS
          GSASGSSG
GSSASASKGSAGASSN
GSVAGASKGSAGASSG
GSSASASKGSAGASSG
SSTAVASKGSAGASSK
GSSASATKGSAGASSC
GSTAGASKGAAGASSN
GSSASASKGSAGASSG
GSTAGASKGSAGASSN
GSSATATKGSAGASSG
NSTAVASKGSAGASSN
GSSASASKGSAGASSQ
GSSASATKGSAGATSN
GSSAVASKGSAGAASG
NSTASATKGSSSASSN
GSSAGATKDGAGAASN
GSTAVASKGSAGAASG
NSTATASKGSAGASSN
GSSATATKGSAGATSN
GSSAVASKGSAGASSG
NSTASASKGSAGASSN
GSSASASKGSAGATSA
GSSAVASKGSAGASSG
NSTASASKGSAGASSN
GSSATASKGSAGASSG
SSSASASKGSAGATSA
GSSAVASKGSAGASSG
NSTASASKGSAGASSN
GSSASASKGSAGATSA
GSSAAASKGSASASSD
GSSAACDSGESDAVDKANLAAIANIAAAAGKPSGKSAPSCDDYYDYEC
```

Figure S2. Protein sequence of ChryC2. Genbank Accession No.: KY906177.

GCNVIAYPTASCGDSGSGSGSASSGAASGAASGSGAASGSGAASGSGSAAASGAASGSGSAAGSGAASGSGSAAGSG
AASGSGSAAGSGAASGSGSAAGSGAASGSGSGSSSSGSSSSGSSGGCGGGSGSASSG
GSSASATKNSAGASSN
GSSAGASNGSAGASSG
GSSASATKNSAGASSG
SSSAGASNGSAGASSC
GSSATATKNSAGASSG
GSTAGASNGSAGASSG
GSSSSATKNSAGASSN
GSSAGASNGSAGASSG
GSSSSATQNSAGASSN
GSSAGASNGSAGASSG
GSSSSATQNSAGASSN
GSSAGASNGTAGASSD
DSDDCSC

Figure S3 (a). Protein sequence of a transcript variant of ChryC1, ChryC1s1. Genbank Accession No.: KY906179.

GCNVIAYPTASCGDSGSGSGSASSGAASGAASGSGAASGSGAASGSGSAAASGAASGSGSAAGSGAASGSGSAAGSG
AASGSGSAAGSGAASGSGSAAGSGAASGSGSGSSSSGSSSSGSSGGCGGGSGSASSG
GSSASATKNSAGASSN
GSSAGASNGSAGASSG
GSSASATKNSAGASSG
SSSAGASNGSAGASSC
GSSATATKNSAGASSG
GSTAGASNGSAGASSG
GSSSSATKNSAGASSN
GSSAGASNGSAGASSG
GSSSSATQNSAGASSN
GSSAGASNGSAGASSG
GSSSSATQNSAGASSN
GSSAGASNGTAGASSG
GSSSSATKESAGASSN
GSTATASKDSAGASSG
GSSVGATASGAGAASGGSVSSATKNSSAASGASAGSSSAAGSGSASGSSGGSSSGASSGSSDGCGSGGSSGAASGAA
SGSGSASGSGAASGSGAASGSGAASGSGAASGSGAASGSGAASGSGAASGSGSASGLGSAASSGAASSSGSAAGSGS
ASGSGSAASSGAASSSGSAAGSGSASVSGSSDDSDDCSC

Figure S3 (b). Protein sequence of a transcript variant of ChryC1, ChryC1s2. Genbank Accession No.: KY906180. The sequence shown in ochre represents the beginning of the short non-repetitive region which interrupts the two repetitive domains in the full-length construct of ChryC1.

GCNVIAYPTASCGDSGSGSGSASSGAASGAASGSGAASGSGAASGSGSAAASGAASGSGSAAGSGAASGSGSAAGSG
AASGSGSAAGSGAASGSGSAAGSGAASGSGSGSSSSGSSSSGSSGGCGGGSGSASSG
GSSASATKNSAGASSN
GSSAGASNGSAGASSG
GSSASATKNSAGASSG
SSSAGASNGSAGASSC
GSSATATKNSAGASSG
GSTAGASNGSAGASSG
GSSSSATKNSAGASSN
GSSAGASNGSAGASSG
GSSSSATQNSAGASSN
GSSAGASNGSAGASSG
G…
…GSASGSSG
GATSTAGSGSASGSSG
NSSSSAGSGSASGSSG
DSSSSAGSGSASGSSG
SASSSAGSGSASGSSG
GSTSGASSGSASGSSG
GSSSSAGSGSASGASAGSSSAAGSGSASGSSGGSSSGASSGSSDGCGSGGSSGAASGAASGSGSASGSGSASGSGAA
SGSGAASGSGAASGSGAASGSGAASGSGAASGSGAASGSGAASGSGAASGSGAASGSGAASGSGAASGSGAASGSGS
ASGSGSAASSGAASSSGSAAGSGSASGSGSSDDDSDDCSC

Figure S3 (c). Partial protein sequence of a transcript variant of ChryC1, ChryC1s3.

GCNVIAYPTASCGDSGSGSGSASSGAASGAASGSGAASGSGAASGSGSAAASGAASGSGSAAGSGAASGSGSAAGSG
AASGSGSAAGSGAASGSGSAAGSGAASGSGSGSSSSGSSSSGSSGGCGGGSGSASSG
GSSASATKNSAGASSN
GSSAGASNGSAGASSG
GSSASATKSSAGASSG
SSSAGASNGSAGASSC
GSSATATKNSAGASSG
GSTAGASNGSAGASSG
GSSSSATKNSAGASSN
GSSA…
…SGSASGSSG
GSASGASCGSASGSSG
DSSSSAGSGSASGSSG
GASSSAGSGSASGSSG
GSTSGASSGSASGSSG
GSSYSAGSGSASGASAGSSSAAGSGSASGSSGGSSSGASSGSSDGCGSGGSSGAASGAASGSGSASGSGSASGSGAA
SGSGAASGSGAATGSGAASGSGAASGSGAASGSGAASGSGAASGSGSASGSGSAASSGAASSSGSAAGSGSASGSES
AASSGAASSSGSAAGSGSASGSGSSDDSDDCSC

Figure S3 (d). Partial protein sequence of a transcript variant of ChryC1, ChryC1s4.

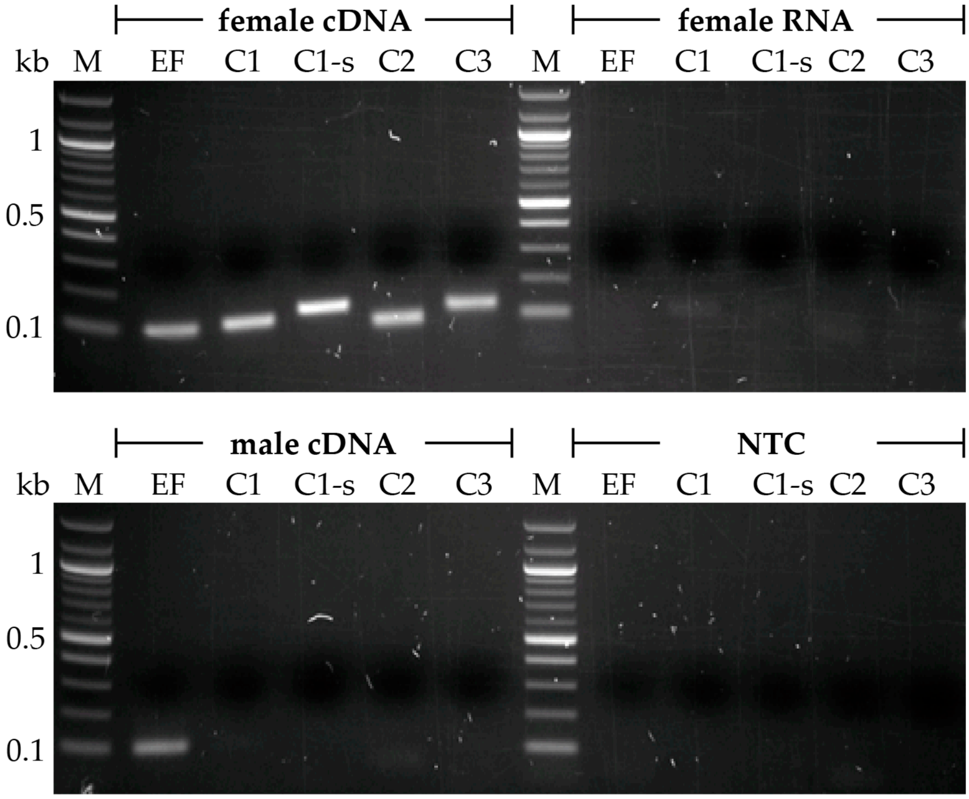

Figure S4. 1.5% (w/v) agarose gel of qPCR products. M: Marker, EF: Elongation factor 1α, C1: ChryC1, C1-s: ChryC1s2, C2: ChryC2, C3: ChryC3, NTC: No template control. Each lane was loaded with 8 μl of qPCR-sample.

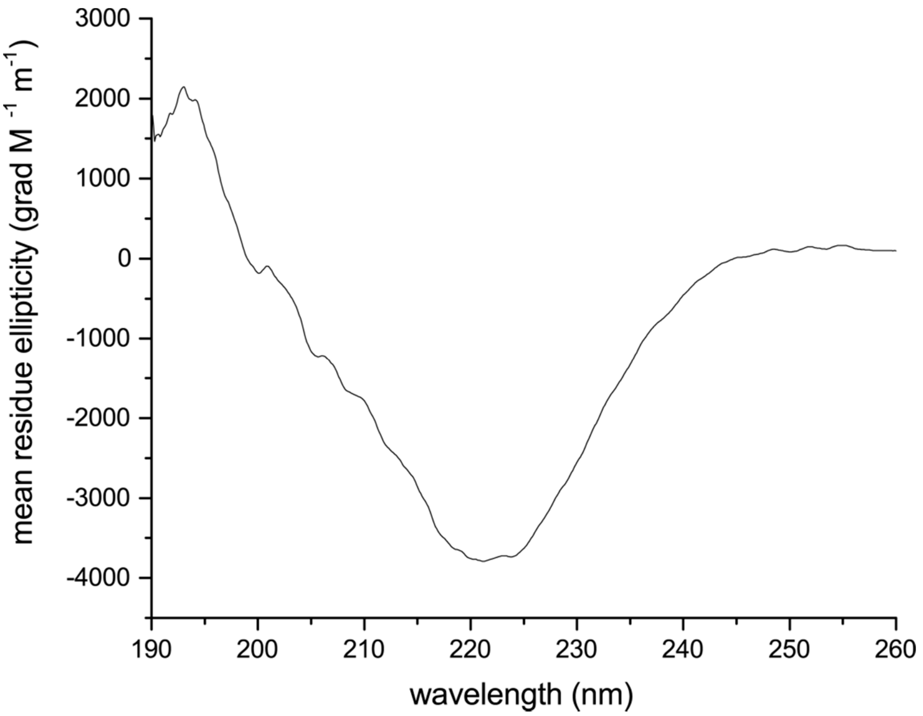

Figure S5. CD spectrum of self-assembled ChryC2 fibrils. Measured in 10 mM sodium phosphate, pH 7.5.

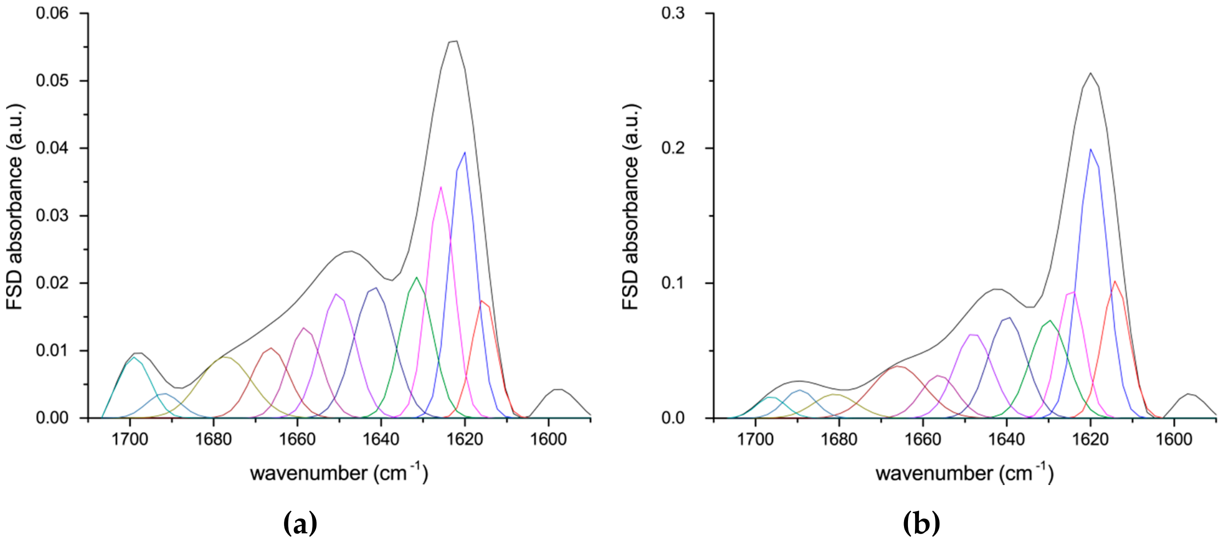

Figure S6. Fourier self-deconvolution (FSD) of an exemplary FTIR spectrum of **(a)** a dried ChryC1 film and **(b)** a wet ChryC1 film after incubation in D_2O.

- Insecta
  - Dicondylia
    - Pterygota
      - Neoptera
        - Endopterygota
          - Neuropterida
            - Neuroptera
              - Chrysopidae
                - Chrysoperla
                  - Chrysoperla adamsi
                  - Chrysoperla agilis
                  - Chrysoperla calocedrii
                  - **Chrysoperla carnea**
                  - Chrysoperla furcifera
                  - Chrysoperla mediterranea
                  - Chrysoperla nipponensis
                - Mallada
                  - Mallada albofacialis
                  - Mallada basalis
                  - Mallada boninensis
                  - Mallada clavatus
                  - Mallada desjardinsi
                  - Mallada krakatauensis
                  - **Mallada signata**

Figure S7. Phylogenetic tree of lacewings according to NCBI taxonomy. The two species discussed in this study are indicated in bold.
